# Elevated hemoglobin A1c Is Associated with Carotid Plaque Vulnerability: Novel Findings from Magnetic Resonance Imaging Study in Hypertensive Stroke Patients

**DOI:** 10.1038/srep33246

**Published:** 2016-09-15

**Authors:** Beibei Sun, Huilin Zhao, Xiaosheng Liu, Qing Lu, Xihai Zhao, Jun Pu, Jianrong Xu

**Affiliations:** 1 Department of Radiology, Renji Hospital, School of Medicine, Shanghai Jiao Tong University, 160 Pujian Road, Shanghai, China; 2 Center for Biomedical Imaging Research, Department of Biomedical Engineering, Tsinghua University School of Medicine, Beijing, China; 3 Department of Cardiology, Renji Hospital, School of Medicine, Shanghai Jiao Tong University, 160 Pujian Road, Shanghai, China

## Abstract

The association between hemoglobin A1c (HbA1c) level and carotid plaque vulnerability has been rarely studied by magnetic resonance imaging (MRI). The present study of MRI-identified carotid atherosclerotic lesions in hypertensive patients with acute stroke therefore sought to determine the associations between HbA1c level and plaque morphological and compositional characteristics and acute cerebral infarction (ACI) severity. Eighty hypertensive patients with acute stroke were enrolled; stratified into high (≥6.5%) and low (<6.5%) HbA1c groups; and underwent carotid and brain MRI to assess carotid plaque features and ACI volume in the region supplied by the internal carotid artery (ICA) in the symptomatic side. Plaque burden [percent wall volume (PWV), max wall thickness (max-WT)] and lipid-rich necrotic core (LRNC) were larger in the high as compared to the low HbA1c group. High HbA1c was an independent risk factor for the presence of plaque (odds ratio [OR] = 3.71) and LRNC plaque (OR = 7.08). HbA1c independently correlated with ACI severity among patients with ICA region cerebral infarction and carotid plaque. Our study suggested that an elevated HbA1c may have an adverse effect on carotid plaque vulnerability especially those with larger LRNC volumes in hypertensive stroke patients, which might exacerbate the severity of ACIs.

As a systemic disease of blood vessels, atherosclerosis from onset through progression and development of clinical complications such as stroke and myocardial infarction^1^,^2^,^3^ is associated with multiple risk factors, among which arterial hypertension^4^ and diabetes mellitus (DM)^5^,^6^ are prominent. Glycosylated Hemoglobin A1c (HbA1c) is a stable index of chronic glycemic status which identifies the average plasma glucose concentration during the past 8–12 weeks^7^, and has been recommended as an optimal method for diagnosing DM. A growing body of evidence has documented a positive relationship between elevated HbA1c levels and an increased risk for cadiovascular disease (CVD) and mortality in patients with or without DM^8^,^9^; also, both low and high mean HbA1c values have been associated with increased all-cause mortality and cardiac events in diabetes patients^10^,^11^. Therefore, HbA1c appears better fit than DM in determining cardiovascular risk.

Ischemic stroke, a major contributor to mortality and disability worldwide^12^,^13^,^14^,^15^,^16^, is significantly associated with carotid atherosclerotic plaques with unstable features such as a large lipid-rich necrotic core (LRNC), intraplaque hemorrhage (IPH), or fibrous cap rupture (FCR)^17^. Using carotid duplex ultrasound, Monica *et al*.^18^ and Lone *et al*.^19^ have found that HbA1c was significantly associated with carotid intima media-thickness (cIMT) and carotid plaques prevalence in non-diabetic patients. However, carotid ultrasound cannot accurately identify vulnerable plaque and characterize tissue composition of human carotid plaque. Magnetic resonance imaging (MRI) can accurately assess both carotid plaque compositional characteristics (i.e., lipid-rich necrotic core and intraplaque hemorrhage) and acute cerebral infarction (ACI) volume in the region supplied by the carotid artery (ICA)^20^,^21^. However, scarce data were available on the association between HbA1c levels and MRI-identified carotid plaque vulnerability and ACI severity in stroke patients. The present study of MRI-identified carotid atherosclerotic lesions in hypertensive patients with acute stroke therefore sought to determine the associations between HbA1c level and plaque morphological and compositional characteristics and ACI severity.

## Results

Between September 2009 and July 2010, 84 subjects meeting the inclusion criteria were recruited for this study. Four subjects were excluded due to overall poor carotid artery image quality. Of the remaining 80 hypertensive patients with acute stroke, 34 (42.5%) had history of type 2 DM, 50 (62.5%) had acute cerebral infarction in ICA territory. The percentage of patients with presence of plaque was 63.8% (51/80) in symptomatic side and 38.7% (31/80) in asymtomatic side.

### Comparison of carotid symptomatic plaque characteristics between the high and low HbA1c groups

According to published criteria^22^, the 80 patients were divided into those with HbA1c < 6.5% (n = 51; low) and those with HbA1c ≥ 6.5% (n = 29; high) with mean HbA1c levels at baseline of 5.34 ± 0.67% and 7.35 ± 0.83%, respectively (P < 0.001). The baseline demographic, clinical characteristics and laboratory findings of these two groups are shown in Table 1. There were no differences in the incidence of classical cerebrovascular risk factors between the two groups except type 2 DM (P < 0.001) and dyslipidemia (P = 0.044). Also, patients with high HbA1c had higher LDL-C (P = 0.042), TC (P = 0.014) as well as higher Scr (P = 0.022) and hs-CRP (P < 0.001). There were no significant differences in other characteristics between these two groups.

The characteristics of the 51 MR-identified plaques in symptomatic carotid arteries in the high and low HbA1c groups are presented in Table 2. The plaque prevalence was higher in patients in the high HbA1c group than in those in the low HbA1c group (86.2% vs 51.0%, P = 0.002). Parameters of plaque burden such as percent of luminal stenosis, max WT, PWV of hypertensive patients with high HbA1c were significantly greater than those of patients in the low HbA1c group. Furthermore, patients with high HbA1c had higher prevalence of American Heart Association (AHA) IV-V type plaque (69.0% vs 39.2%, P = 0.011), LRNC plaque (86.2% vs 45.1%, P < 0.001) and LRNC% > 40% plaque (31.0% vs 3.9%, P = 0.001) than those in the low HbA1c group. There were no significant differences between groups with regard to the presence of IPH, CA, FCR or IPH%, CA%.

### Associations between HbA1c level and carotid symptomatic plaque characteristics

**Figure 1:**
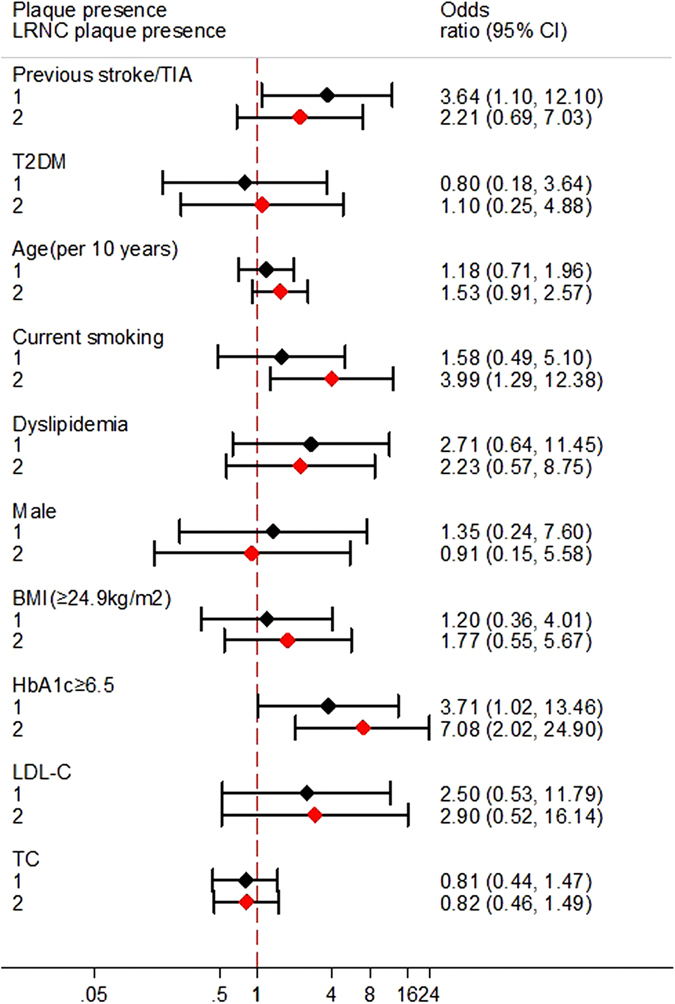
Independent predictors of the presence of plaques (1) and lipid-rich necrotic core (LRNC) plaques (2) in hypertensive stroke patients (n = 80).

In multivariate logistic regression analysis (Fig. 1), high HbA1c level [odds ratio (OR) = 7.08, 95% confidence interval (CI) = 2.02–24.90, P = 0.002] and current smoking (OR = 3.99, 95% CI = 1.29–12.38, P = 0.017) were independent risk factors for the presence of LRNC plaques. High HbA1c (OR = 3.71, 95% CI = 1.02–13.46, P = 0.046) and previous stroke/TIA (OR = 3.64, 95% CI = 1.10–12.10, P = 0.035) were independent factors for plaque presence.

**Figure 2:**
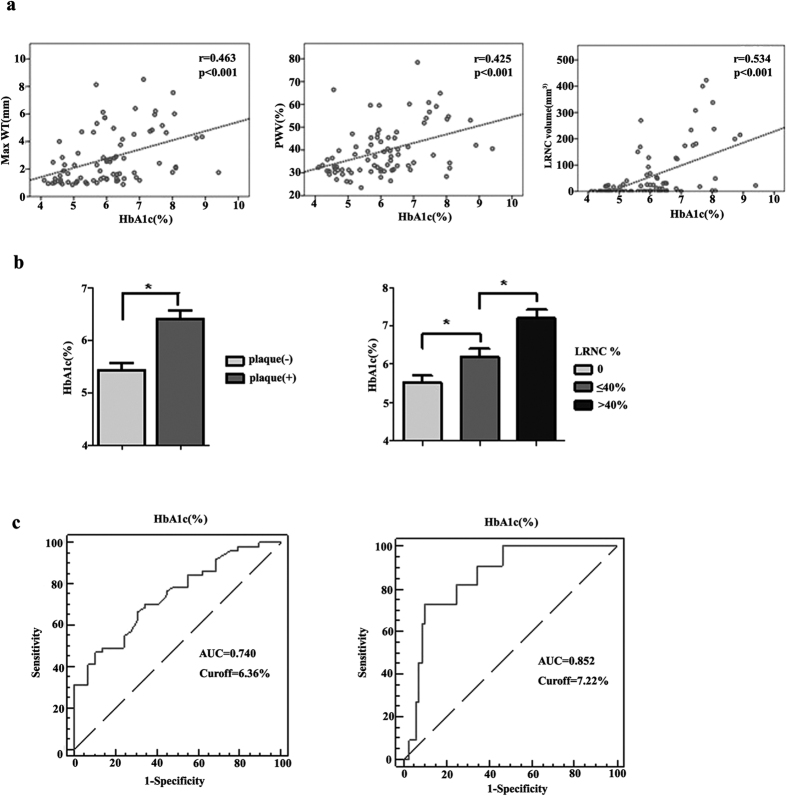
Correlation between HbA1c and carotid plaque features. (**a**) Scatter plot of HbA1c level and plaque Max WT, PWV and LRNC volume; (**b**) HbA1c levels for different plaque features; (**c**) ROC curve to determine the optimal cutoff value for plaque presence(*left*) and large LRNC plaque presence(*right*). *p < 0.05.

Figure 2a illustrates the correlation scatter plot of HbA1c level with PWV, max WT and LRNC volume. HbA1c was positively associated with plaque burden: max WT (r = 0.463, P < 0.001), PWV (r = 0.425, P < 0.001), as well as the LRNC volume (r = 0.534, P < 0.001).

In addition, HbA1c level was higher in patients with carotid plaque than without plaque (6.40 ± 1.20% vs 5.42 ± 0.85%, P < 0.001). There was also an increase tendency of LRNC% of plaque lesion with elevated HbA1c levels (Fig. 2b). Optimal threshold of HbA1c to predict the presence of different plaque features are shown in Fig. 2c. The Receivers operating characteristic (ROC) showed that 6.36% was the optimal threshold of HbA1c level to predict the presence of symptomatic plaque. The use of this value yielded a sensitivity of 65.5% and a specificity of 70.6% [Area under the receivers operating characteristic (AUC) = 0.740] (Fig. 2c, left). The ROC curve also showed that 7.22% was the optimal cutoff value of HbA1c level to predict the presence of larger LRNC (%volume of LRNC > 40%) (AUC = 0.852, sensitivity, 72.7%; specificity, 89.9%) (Fig. 2c, right).

### Association between HbA1c and acute cerebral infarction severity

**Figure 3:**
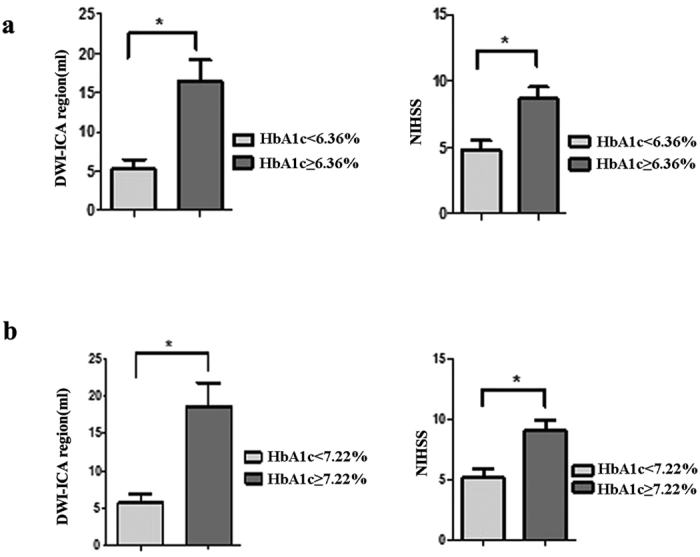
DWI hyperintensity volume in ICA region (DWI-ICA region) and National Institutes of Health Stroke Scale (NIHSS) score between different HbA1c groups. (**a**) HbA1c < 6.36% and HbA1c ≥ 6.36% group; (**b**) HbA1c < 7.22% and HbA1c ≥ 7.22% group. *p < 0.05.

Among 31 patients with DWI-identified ACIs and MRI-identified carotid plaque in the symptomatic side, NIHSS, indicating more severe neurological symptomatology, was higher in patients with HbA1c ≥6.36% (n = 14) than in those with HbA1c <6.36% (n = 17) (P < 0.05). As for imaging findings, ACI volume was also larger in patients with HbA1c ≥6.36% patients than in those with HbA1c <6.36% patients (16.38 ± 10.48 vs 5.24 ± 5.15, P = 0.002) (Fig. 3a).

Using the optimal cutoff value of HbA1c of 7.22% to predict larger LRNC: 11 patients had HbA1c ≥7.22%, and 20 patients <7.22%, with a significantly different NIHSS (P < 0.05) and ACIs volume in ICA region (18.66 ± 10.35 vs. 5.65 ± 5.30, P = 0.002) (Fig. 3b).

**Figure 4:**
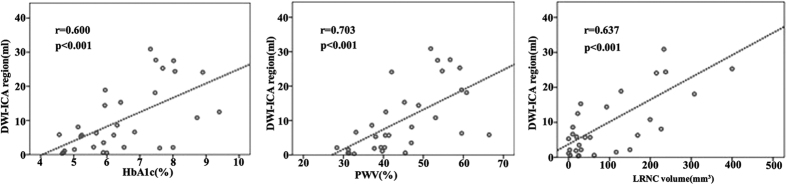
Scatterplots depicting the relationship of the significant predictors with the cerebral infarction severity.

**Figure 5:**
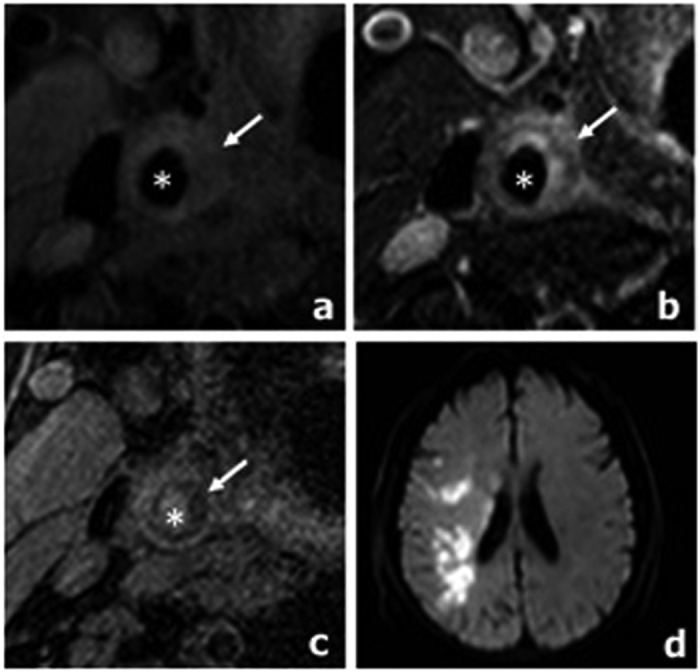
Representative MR images of a subject with high level HbA1c (HbA1c = 7.8%). (**a–c**) An atherosclerotic plaque with large LRNC (arrow) is detected in the right carotid artery (star): iso-intensity on T1-weighted image (**a**); hypointensity on corresponding T2-weighted image (**b**); hypo-intensity on MP-RAGE image (**c**). (**d)** Cerebral DWI demonstrates ACIs (hyperintensity) at right hemisphere.

When we explored the association between ACI severity and HbA1c level, univariate regression and multiple linear regression analysis using a stepwise selection process was performed to adjust for the variables shown to be likely to be independently associated with ACI. Table 3 showed that in univariate regression analysis, HbA1c, luminal stenosis, PWV, max WT, LRNC volume and IPH volume all closely correlated with the volume of ACI in hypertensive stroke patients with symptomatic carotid plaque. In multiple linear regression after adjustment for demographic factors such as age, sex, BMI and cerebrovascular risk factors such as current smoking, Type 2 DM, dyslipidemia, previous stroke/TIA, HbA1c (β = 0.37, P = 0.003), LRNC volume (β = 0.33, P = 0.028), PWV (β = 0.34, P = 0.016) as well as BMI (≥24.9 kg/m^2^) (β = 0.23, P = 0.034) were independently related to the severity of ACI. The relationships between HbA1c level, LRNC volume and PWV with ACI volume are shown in Fig. 4. Figure 5 shows an example of carotid and brain MR images in a subject with HbA1c = 7.8%.

## Discussion

This is a MRI study investigating the relationships among HbA1c, carotid plaque features and the severity of ACI in hypertensive patients with acute stroke. The main findings of the present study are: (1) hypertensive patients with high HbA1c exhibit more MRI-identified carotid LRNC plaques as well as larger plaque burden and larger LRNC% than patients with low HbA1c; (2) high HbA1c level was an independent risk factor for plaque and LRNC plaque presence, and HbA1c level was closely related to carotid plaque characteristics, especially larger LRNC; (3) using the ROC curve analysis, optimal cutoff values were determined for HbA1c to predict MRI determined plaque (HbA1c = 6.36%) and larger LRNC plaque (HbA1c = 7.22%) and (4) multivariate analysis identified HbA1c as a potentially independent predictor of severity of ACI.

HbA1c, which is an established diagnostic marker for diabetes mellitus^23^, also plays a contributory role in the progress of atherosclerosis in both diabetic and non-diabetic individuals. Compared with fasting glycemia, HbA1c is a more stable and accurate parameter of glucose homeostasis, and might offer more advantages in terms of prognostic impact^24^. An epidemiological study of community-dwelling Japanese subjects with glucose intolerance reported that, elevated HbA1c provided superior discrimination for carotid wall thickening compared to 1,5-anhydroglucitol, fasting plasma glucose, and 2-hour postload glucose^25^. Monica *et al*.^18^ found that HbA1c, but not fasting glycemia, was linearly associated with the cIMT in non-diabetics. Using carotid ultrasound, Ulrike *et al*. showed that HbA1c was an independent risk factor for cIMT in long-term survivors of ischemic stroke^26^. Comparing with carotid ultrasound, MRI can accurately characterize plaque morphology and tissue composition of human carotid atherosclerotic plaque^27^. Yuan *et al*. demonstrated that MRI could identify the LRNC and IPH in carotid plaque as well as classification of carotid atherosclerotic lesions with high sensitivity and specificity^20^. The present study found that in hypertensive stroke subjects HbA1c was closely related to the carotid plaque vulnerability on MRI. Patients with high HbA1c exhibit more MRI-identified carotid LRNC plaques as well as larger plaque burden and larger LRNC%. Moreover, multivariate regression analysis identified high HbA1c level as an independent risk factor for LRNC plaque presence.

HbA1c also has been proposed as a reliable tool for identifying individuals at high risk of cardiovascular events with and without DM^28^,^29^. ACI volume on DWI has been found to closely correlate with acute clinical severity and stroke outcomes^30^. Atherothromboembolic processes caused by disruption of high-risk carotid plaque lead to occlusion of large intracranial and extracranial blood vessels causing most acute ischemic strokes^31^, and our previous study also showed that carotid plaque characteristics, particularly PWV and LRNC size were associated with the size of ipsilateral ACIs^17^. In addition, clinical and population-based cohort studies have shown a linear association between HbA1c levels and the risk of incident CVD, including ischemic stroke^32^,^33^. Jia *et al*. reported that abnormal glucose regulation was prominent among Chinese patients with acute stroke, especially in those with atherothrombotic infarction^34^. Wu *et al*. indicated that HbA1c was significantly associated with increased risk for poor outcomes in the first year after acute ischemic stroke^35^. Consistent with the latter studies, in the present study, HbA1c showed a positive relationship with DWI lesion volume in ICA territory, which was closely correlated with severity and prognosis of ischemic stroke outcomes, similar to the relationship observed with coronary heart disease which appears to be continuous over the whole distribution of HbA1c^9^. These findings are consistent with those of a few population studies showing that HbA1c might exhibit a significantly detrimental role even when it was in “normal” or relatively low levels^36^.

In terms of HbA1c predicting ACI extent, several hypotheses have been put forth to explain the causal relationship between hyperglycemia and atherosclerosis^37^, with glycosylation being recognized as one of the important pathways. As one of the end products of glycosylation, HbA1c and its advanced glycation end products can trigger a vicious cycle of oxidative/inflammatory responses which might have a direct role on accelerating the progression^38^,^39^ and rupture of atherosclerotic lesions and adverse cardiovascular events. In addition, other studies have demonstrated that the compositions and volumes of coronary artery plaques were associated with the glycemic control status (HbA1c level) of participants with diabetes^40^. Consistent with the latter reports and hypotheses, in the present study, patients with high levels of HbA1c also had higher levels of inflammatory biomarkers such as hs-CRP, in association with more extensive LRNC composition and larger plaque burden (PWV and max WT). These biological mechanisms of glycosylation may underlie the relationship between HbA1c and the extent of ACI in the ICA territory observed in the present study.

The present study has several limitations. First, because of the generally long MR acquisition time, it was difficult to get sufficient cooperation from patients with severe symptoms. Secondly, this study used a cross-sectional design with a relatively small number of participants. Prospective follow-up studies with larger sample size is required to validate the correlation between HbA1c and carotid atherosclerotic disease progression with the severity of future ACI events. Thirdly, the observational design of the present study may have introduced bias, so adjustment was performed for differences in known risk factors for cerebrovascular disease (e.g., smoking, dyslipidemia and demographic factors) to minimize their effects on plaque features and ACI severity. Finally, we did not measure other diabetes related factors such as fasting glucose, 2-hour postload glucose, fasting insulin and insulin resistance. Further studies are warranted to explore the associations between other diabetes related factors and carotid plaque vulnerability/ACI severity.

## Conclusions

The results of the present study suggest that an elevated HbA1c may have an adverse effect on carotid plaque lesions especially those with larger LRNC volumes and PWV in hypertensive stroke patients with carotid atherosclerosis, which might exacerbate the severity of ACIs in the ICA territory. Our findings indicate that determination of HbA1c levels and characterization of carotid atherosclerotic plaque by MR vessel wall imaging might be useful to better select proper treatment options of stroke subjects.

## Methods

### Participants

All experimental protocols were approved by the Ethic Committee of Shanghai Renji Hospital, and the experimental protocols were performed in accordance with the approved guidelines. Informed consent was obtained from all of the subjects before the study began. The observational study enrolled hypertensive patients with acute ischemic stroke in the anterior circulation who were referred for brain and carotid MRI examination within 1 week after the onset of neurovascular symptoms. Exclusion criteria were: (1) high-risk cardioembolic sources (e.g. paroxysmal atrial fibrillation); (2) other etiologies such as vasculitis, moyamoya disease or cancer-related stroke; (3) patients with intracranial artery stenosis; (4) patients with a severely altered mental status (i.e., coma); and (5) contraindications for MRI. Data from MRI (carotid plaque and cerebral infarction) and medical records including results of neurological examination [National Institutes of Health Stroke Scale (NIHSS) score], laboratory analysis [high-sensitivity C-reactive protein (hs-CRP), serum creatinine (Scr), glomerular filtration rate (GFR), HbA1c], and patient data, including age, sex, BMI, and cerebrovascular risk factors (e.g. T2DM, dyslipidemia, hypertension, current smoking, and a history of ischemic disease), were collected and entered into the study database.

Hypertension was defined as a systolic blood pressure ≥140 mmHg, a diastolic blood pressure ≥90 mmHg, or current treatment with antihypertensive agents. Dyslipidemia was defined as triglyceride (TG) ≥1.7 mmol/L and high-density lipoprotein-cholesterol (HDL-C) <1.03 mmol/L (for male) and <1.29 mmol/L (for female). The patients were defined as having DM based primarily on their blood glucose levels, i.e., either a fasting plasma glucose (FPG) level of ≥7.0 mmol/l or an oral glucose tolerance test (OGTT) result of ≥11.1 mmol/l^41^. HbA1c was measured by high-performance liquid chromatography with a Bio-Rad Diamat device (Richmond, CA). The inter-assay coefficient of variation (CV) was 3.1%, and the intra-assay CV was 2.5%, both within NGSP acceptable limits. The symptomatic carotid artery was defined as that responsible for the neurological symptoms.

### Carotid MRI Protocol

All patients were imaged using a 3.0T MRI scanner (Achieva; Philips Healthcare, Best, The Netherlands) with an 8-channel phased-array carotid coil (Chenguang Medical Technologies, Shanghai, China). A standardized imaging protocol was followed to obtain multicontrast cross-sectional MRI scans, including time of flight (TOF), T1-weighted, T2-weighted, and magnetization-prepared rapid acquisition gradient-echo (MP-RAGE) imaging of the bilateral carotid arteries centered on the bifurcation of the symptomatic carotid artery. The following MRI parameters were used: 3D TOF: TR/TE, 20/5.1 ms; flip angle, 20°; quadruple inversion recovery T1-weighted sequence: TR/TE, 800/10 ms; T2-weighted sequence with multi-double inversion recovery 18: TR/TE, 4000/50 ms; and 3D MPRAGE sequence: TR/TE, 9.2/5.5 ms; and flip angle, 15°. All MRI axial scans were acquired with a section thickness of 2 mm, an FOV of 14 cm × 14 cm, a matrix size of 256 × 256, and an in-plane resolution of 0.54–0.55 mm. The longitudinal coverages of the black-blood (T1-weighted, T2-weighted, and 3D MPRAGE) and bright-blood (3D TOF) sequences were 32 mm (16 sections) and 44 mm (22 sections), respectively. Fat saturation was applied to the acquisition of the black-blood sequences to enhance the tissue contrast between the carotid vessel wall and the surrounding tissues. Maximum-intensity-projection MRA images were reconstructed from the 3D TOF images.

### MRI Interpretation

Two experienced radiologists (QL. and X.L., >5 years of experience in neuroradiology) blinded to the clinical information and carotid MRI scans evaluated the brain DWI images. Each of them finished the whole data analyses separately. Reproducibility was assessed by replicating measurements for 10% of the participants 3 months after the initial evaluation. The intraclass correlation coefficient for inter-observer reproducibility was 0.92[0.75 to 0.95] for the DWI hyperintensity volume. Acute cerebral infarctions (ACIs) were defined as hyperintense regions on the DWI images and hypointense regions on the diffusion coefficient map. The presence and volume of the ACIs were determined using the image of the maximum contrast between the lesion and the normal brain regions (i.e., the DWI with the highest b value) using a Philips MR workstation (Extended MR Workspace 2.6.3.1, Philips Medical System, Best, Netherlands). DWI lesion volumes were assessed using the affected slices with hyperintense areas visible from the b = 1,000 mm/s^2^ images. The neurologists paid particular attention to the typical locations of bilateral artifacts and produced apparent diffusion coefficient maps as necessary to identify positive DWI lesions. The sum of the volumes of the ACIs in the ICA blood-supplying territories was recorded for the hemisphere on the symptomatic side of each patient.

Two trained reviewers (X.Z. and H.Z., ≥3 years of experience in carotid plaque imaging) interpreted the carotid MRI scans from the symptomatic side via consensus using custom-designed software (CASCADE^42^, Seattle, WA, USA). Image quality was rated per axial location on a four-point scale (1, poor; 2, marginal; 3, good; 4, excellent) depending on the overall signal-to-noise ratio and the clarity of the vessel wall boundaries. Slices with an image quality <2 were excluded from review. Morphological measurements, including the maximum wall thickness (max WT) and percent wall volume (PWV) were obtained for each artery. The presence or absence and the volumes of each carotid plaque component (e.g., the LRNC, CA, and IPH) were identified based on previously published criteria validated by histology^21^. Carotid atherosclerotic plaque was defined as lesions with presence of any plaque component (e.g., CA, LRNC, FCR or IPH) on MR images. Percent volume of component (component%) = component volume/plaque volume. According to the modified AHA criteria^43^, type IV–V lesion was assigned to plaques characterized by a lipid or necrotic core surrounded by fibrous tissue with possible calcification; and type VI lesion to complex plaque with possible surface defect, hemorrhage, or thrombus. Type IV–VI lesions were identified as vulnerable plaques. The luminal stenosis of the symptomatic carotid arteries was measured using the NASCET criteria (percent stenosis = 100% × [1 − the luminal diameter at the point of maximal narrowing/the diameter of the normal distal internal carotid artery])^44^ using a Philips MR workstation.

### Statistical Analyses

All analyses were performed using R 2.11.0 (R Development Core Team, 2010). Continuous and categorical data are presented as means ± SDs and percentages, respectively. The continuous variables were compared via the independent-samples t-test when normally distributed or the Mann-Whitney U test when non-normally distributed. The categorical variables were compared using the chi-square test. A logistic regression was performed to assess the associations between the presence of plaques, LRNC and HbA1c after adjustment for other cardiovascular risk factors and demographic factors. ROC curves were generated from multiple sensitivity/specificity pairs. The optimal cutoff point was defined that on the ROC curve closest to (0,1)^45^. Generalized linear models using a stepwise selection process were used to determine the association between HbA1c, carotid plaque features and ACI severity. Multivariate regression analysis was adjusted for confounders and variables that were significant in univariate analysis (p < 0.2). All tests were 2-tailed, and P values < 0.05 were considered significant.

## Additional Information

**How to cite this article**: Sun, B. *et al*. Elevated hemoglobin A1c Is Associated with Carotid Plaque Vulnerability: Novel Findings from Magnetic Resonance Imaging Study in Hypertensive Stroke Patients. *Sci. Rep.* **6**, 33246; doi: 10.1038/srep33246 (2016).

## Figures and Tables

**Table 1:** Baseline data of hypertensive patients according to HbA1c level (n = 80).

Variables	HbA1c < 6.5% (n = 51)	HbA1c ≥ 6.5% (n = 29)	*P value*
Age, yr	61.65 ± 11.27	66.17 ± 9.98	0.076
Male gender	37 (72.5%)	22 (75.9%)	0.512
BMI, kg/m^2^	24.56 ± 2.99	24.67 ± 2.86	0.868
T2DM	10 (19.6%)	24 (82.80%)	<0.001
Dyslipidemia	9 (17.60%)	11 (37.90%)	0.044
Current smoker	27 (52.90%)	17 (58.60%)	0.624
Previous stroke/TIA	15 (29.40%)	11 (37.90%)	0.434
HDL-C, mmol/L	1.12 ± 0.25	1.14 ± 0.29	0.815
LDL-C, mmol/L	2.83 ± 0.73	3.21 ± 0.92	0.042
TG, mmol/L	1.65 ± 1.07	2.02 ± 0.96	0.130
TC, mmol/L	4.61 ± 0.91	5.25 ± 1.12	0.014
Scr (μmol/L)	68.81 ± 15.69	83.71 ± 27.32	0.022
GFR (ml/min)	57.19 ± 18.59	68.50 ± 32.16	0.244
Hs-CRP (mg/L)	3.23 ± 3.59	8.88 ± 7.73	<0.001

Note: Continuous data are presented as mean ± SD; Categorical data are presented as n (%). T2DM, Type 2 Diabetes mellitus; BMI, Body Mass Index; LDL-C, low-density lipoprotein cholesterol; HDL-C, high- density lipoprotein cholesterol; TG, triglyceride; TC, total cholesterol; HbA1c: Hemoglobin A1c; Hs-CRP: High-sensitivity C-reactive protein; Scr: serum creatinine; GFR: glomerular filtration rate.

**Table 2:** MR vessel wall imaging findings of symptomatic carotid plaques in hypertensive patients with different HbA1c levels.

Variables	HbA1c < 6.5% n = 51	HbA1c ≥ 6.5% n = 29	*P*
Plaque general features			
Plaque presence [n](%)	26 (51.00%)	25 (86.20%)	0.002
Plaque burden			
Luminal Stenosis (%)	14.93 ± 20.42	34.29 ± 30.91	0.003
Max WT (mm)	2.33 ± 1.68	3.62 ± 2.14	0.004
PWV (mm^3^)	36.40 ± 8.93	45.06 ± 12.59	0.001
Plaque vulnerable parameters			
AHA type IV-V [n](%)	20 (39.20%)	20 (69.00%)	0.011
AHA type VI [n](%)	3 (5.90%)	5 (17.20%)	0.131
LRNC prevalence [n](%)	23 (45.10%)	25 (86.20%)	<0.001
LRNC% > 40% prevalence^a^	2 (3.90%)	9 (31.00%)	0.001
LRNC%^a^^,^^b^	11.54 ± 16.07	32.43 ± 33.92	0.033
IPH prevalence [n](%)	3 (5.90%)	5 (17.20%)	0.131
IPH%a,^b^	3.97 ± 13.69	6.36 ± 14.59	0.405
FCR prevalence [n](%)^c^	1 (2.00%)	1 (3.4%)	—
CA prevalence [n](%)	17 (33.30%)	15 (51.72%)	0.107
CA%^a^,^b^	7.82 ± 16.94	5.96 ± 10.65	0.954

Note: PWV, percent wall volume; Max WT, max wall thickness; LRNC, lipid-rich necrotic core; IPH, intraplaque hemorrhage; FCR, Fibrous cap rupture; CA, calcification.

^a^Component% = corresponding component volume/plaque volume.

^b^Only including those with plaque present.

^c^Too few patients for chi-square analysis. Continuous data are presented as mean ± SD; Categorical data are presented as n (%).

**Table 3:** Correlation between HbA1c and cerebral infarction severity in hypertensive stroke patient with DWI hyperintensity and plaque (n = 31).

Variables	DWI Hyperintensity Volume in ICA Territory^a^			
	Univariate		Multivariate	
	β	*P*	β	*P*
Age (per 10 years)	0.38	0.034		
Male	−0.14	0.147		
Current smoker	0.19	0.319		
BMI (>24.9 kg/m^2^)	0.23	0.217	0.23	0.034
Dyslipidemia	0.15	0.433		
Previous stroke/TIA	0.29	0.120		
T2DM	0.21	0.252		
HbA1c	0.55	0.001	0.37	0.003
Luminal stenosis (%)	0.54	0.002		
PWV (mm^3^)	0.70	<0.001	0.34	0.016
Max WT (mm)	0.51	0.001		
LRNC Volume (mm^3^)	0.69	<0.001	0.33	0.028
IPH Volume (mm^3^)	0.44	0.013		
CA Volume (mm^3^)	0.06	0.755		

^a^Only including those with plaque present and DWI hyperintensity in ICA territory. β indicates standardized coefficient.
